# Research progress in artificial intelligence for brain metastases

**DOI:** 10.3389/fmed.2025.1643850

**Published:** 2025-09-30

**Authors:** Dongxiang Wang, Wei Wang, Tong Li, Chenqi Liang, Xia Zhao

**Affiliations:** ^1^Medical Imaging, Faculty of Radiology, Shandong First Medical University, Tai'an, Shandong, China; ^2^Clinical Medicine, Faculty of Clinical and Basic Medicine, Shandong First Medical University, Jinan, Shandong, China; ^3^Department of Nuclear Medicine, Shandong Provincial Hospital Affiliated to Shandong First Medical University, Jinan, Shandong, China; ^4^Department of Radiology, Shandong University of Traditional Chinese Medicine Affiliated Hospital, Jinan, China

**Keywords:** brain metastases, MRI, deep learning, machine learning, CT

## Abstract

As artificial intelligence (AI) continues to evolve, its integration into medical practice is becoming increasingly prominent, particularly in the field of neuro-oncology. This review examines the application of AI—specifically machine learning (ML) and deep learning (DL)—in the imaging evaluation of brain metastases (BM). A systematic search of PubMed was conducted to identify relevant studies published within the past 5 years. The retrieved literature was categorized and analyzed according to three key clinical tasks: segmentation, differential diagnosis, and prognostic prediction. We first outline the capabilities of AI in the automatic detection and segmentation of BM using advanced imaging techniques. Subsequently, we synthesize evidence on how AI aids in distinguishing BM from other intracranial structures and lesions. Finally, we discuss the emerging role of AI in predicting disease prognosis and the development of new metastatic abnormalities. Current evidence suggests that AI not only enhances diagnostic efficiency and reproducibility but also provides clinically meaningful insights that support personalized treatment planning. Importantly, the integration of AI into neuro-oncological imaging remains at a nascent stage, indicating substantial potential for future growth and refinement in both technical performance and clinical applicability.

## 1 Introduction

BM represent secondary malignant lesions that disseminate to the brain from primary extracranial tumors via hematogenous or lymphatic pathways. With continued improvements in cancer patient survival, the incidence of BM has risen significantly, currently affecting between 10% and 40% of all cancer patients (1). The most frequent primary malignancies leading to BM include lung cancer, breast cancer, melanoma, renal cell carcinoma, and colorectal cancer. Notably, non-small cell lung cancer (NSCLC) alone accounts for BM in approximately 28.6% of cases (2).

Artificial intelligence (AI), a rapidly advancing discipline within computer science, aims to emulate human cognitive functions to address complex problems. A core goal of AI is to develop systems capable of autonomous reasoning and decision-making, with performance levels that can meet or exceed human expertise in specific domains. In healthcare, AI algorithms are increasingly being deployed to improve diagnostic precision, prognosticate clinical outcomes, accelerate drug development, and enhance the efficiency of large-scale data analysis in biomedical research.

**Figure 1:**
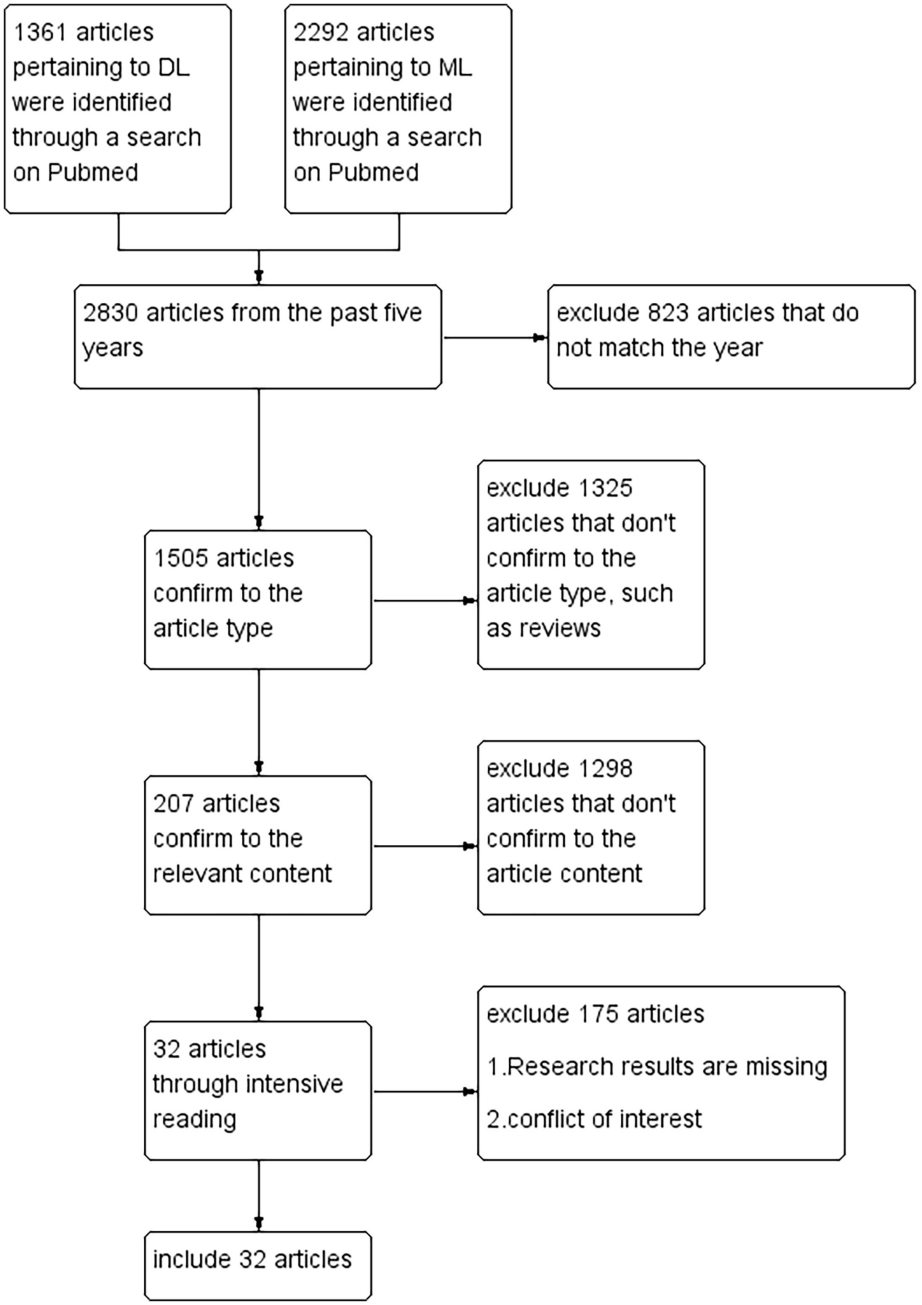
Specific steps for filtering articles. A total of 3,653 articles on AI were collected. Eight hundred and twenty-three articles were excluded based on age, but 1 prospective article was retained. One thousand three hundred and twenty-five articles were excluded based on article type, and 1,298 articles were excluded based on content relevance. Finally, 32 articles were retained through intensive reading.

Through a comprehensive review and synthesis of current literature, we have identified and summarized key advancements in AI applications for BM management. An overview of this process is illustrated in Figure 1.

## 2 Application of AI in BM detection and segmentation

The integration of AI into the detection and segmentation of BM reflects a critical alignment between unmet clinical demands and technological feasibility. Current evidence indicates that AI holds considerable promise for improving diagnostic accuracy, reducing missed diagnoses, and supporting more precise treatment planning. Research efforts are primarily concentrated on high-resolution MRI, while exploration of emerging imaging techniques—as well as the fusion of multimodal imaging data—represents a major direction for future innovation. Key developments in this domain are summarized in Table 1.

**Table 1:** Summary of diagnostic models for brain metastases.

**Models**	**DeepMedic-based CAD**	**3D V-Net CNN**	**nnU-Net DLS**	**Deep learning ensemble ModelnnDetection nnU-Net DeepMedic+**	**CE + NECT modle vs. CECT model**	**Expand nnU-Net (ADS/ADL)**	**2.5D DeepLabv3 Network + Input level dropout layer**	**SimU-Net**
Authors	Fairchild et al.	Hsu et al.	Park et al.	Bartosz Machura et al.	Hidemasa Takao et al.	Youngjin Yoo et al.	Darvin Yi et al.	Yonny Hammer et al.
Number of patients	135	511	101+243	275	116	2092	100+57	169+57
Exclusion criteria	After brain treatment and small cell carcinoma	No brain metastases or other brain tumors	No follow-up MRI patient, non-primary lung cancer source	Lack of required sequence images; presence of motion artifacts	Lesions that have not been confirmed by radiologists; Missing image	Only those with brain parenchymal metastases measuring ≥ 2 mm; Lesions adjacent to the surgical bed	History of surgery or radiation therapy; missing image	Non metastatic cancer; Recently underwent craniotomy surgery; Lack of continuous scanning data
Conclusion	Sensitive to small, difficult-to-detect metastases (< 3 mm sensitivity, 79%), with a low false-positive rate (2 per patient).	Segmentation performance (DSC 0.76), false positive rate of 2.4 per patient	DLS enhances repeatability (CCC 0.918), decreases reading time (9.6 seconds), and has a sensitivity of 90.2%	Recall rate: 0.664; Supports automatic tracking of disease progression	PPV increased to 44.0%; significantly reducing false positives.	Sensitivity for detecting small lesions (< 0.1 cm 3) is 0.824; Average DSC 0.758; HD95 1.45 mm	The best performance is achieved with small datasets; robustness to missing pulse sequences.	Superior to single scan models in longitudinal detection, the accuracy of automatically matching lesion changes is 100%
Limitations	Single-center data, utilizing solely T1-weighted MRI	Iodine contrast agent, overestimation of lesion volume, and the false positive impact on fully automated applications.	Applicable only to lung cancer patients; not applicable to other conditions.	The number of patients is limited; the performance of large lesions is unknown.	Not compared with radiologists; a small amount of data	There are observer differences in the true contour; the quality of lesion annotation is limited.	Data may exhibit protocol differences; the introduction of bias; small sample size may limit generalizability	Single-center data; low performance in identifying small lesions; limiting clinical applicability

This table provides a comparative summary of segmentation models for brain metastases. It is noteworthy that nnU-Net demonstrates superior stability and robustness compared to alternative models. Future developments are expected to prioritize multimodal data fusion, reduction of false positives, enhancement of interpretability, and validation of clinical utility. These advancements are anticipated to facilitate its translation from a research tool into routine clinical practice.

### 2.1 Detection and segmentation in single sequence MRI

BM represent a frequent and serious complication of systemic cancer, profoundly affecting patient survival and quality of life. Early and accurate detection of BM is essential for formulating effective radiation therapy strategies. However, manual detection and segmentation remain substantially challenging due to the characteristically small size, high multiplicity, and ill-defined borders of metastatic lesions.

DL-based approaches offer promising solutions to these limitations. The integration of architectures such as the Single Shot MultiBox Detector (SSD) has been shown to markedly enhance detection performance in DL systems (3).

**Figure 2:**
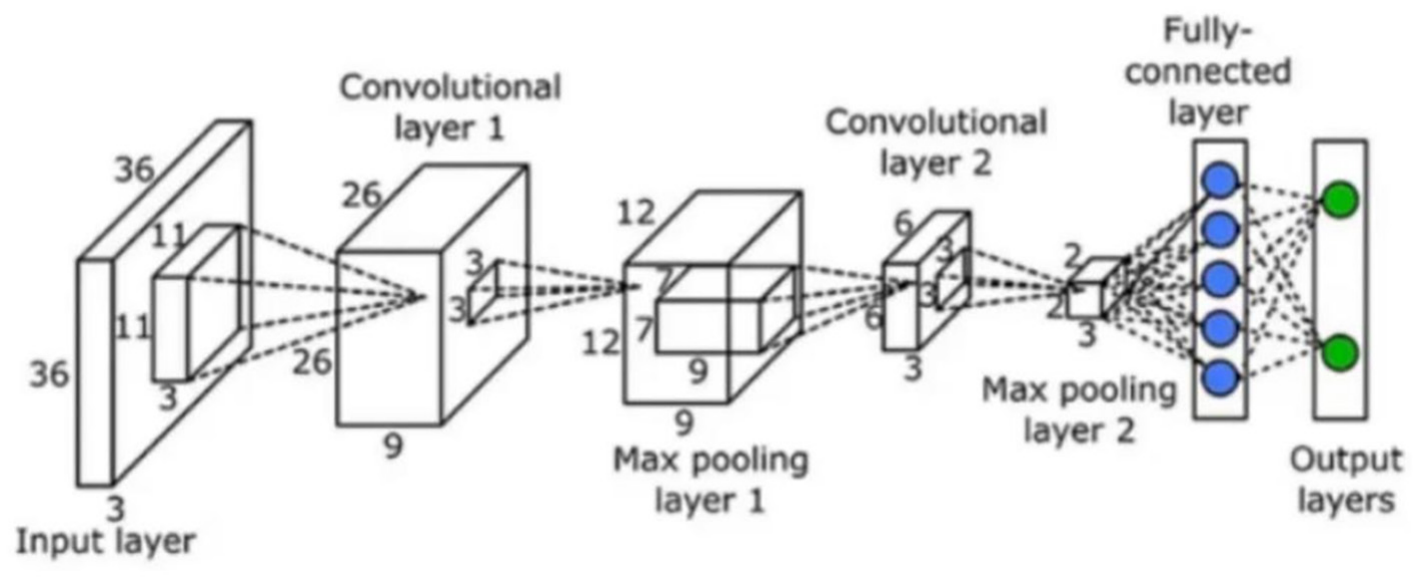
Schematic diagram of the CNN model architecture. The process begins with inputting the target image. The second step involves extracting features from the target using convolutional kernels. Once extracted, these features undergo pixel-level processing. The third step further compresses the features through max pooling, which helps retain the most salient characteristics and organizing the pooled features into a 3 × 3 grid. In step four, the feature map is flattened, converting the 3 × 3 patches into a one-dimensional array. Step five consists of feeding this flattened data into a fully-connected layer. Finally, the output result is generated.

The U-Net architecture, a widely adopted convolutional neural network (CNN) for biomedical image segmentation (illustrated in Figure 2), excels at combining high-resolution contextual details with semantically rich features, leading to significant gains in segmentation accuracy. Further refinements through novel algorithmic integrations can augment the model's precision (4). The incorporation of longitudinal timeline analysis, in addition to cross-sectional evaluation, has also been demonstrated to improve BM detection rates (5).

Moreover, the nnU-Net (Extended U-Net Model)—an adaptive framework designed for medical image segmentation—can be optimized through the incorporation of two specialized modules: Adaptive Data Sampling (ADS) and Adaptive Dice Loss (ADL). These enhancements have proven effective in substantially elevating detection performance (6).

### 2.2 Combining multi-sequence MRI for brain metastasis detection and segmentation

BM exhibit distinct and often complementary imaging features across different MRI sequences, a characteristic that has prompted most current DL models—including those discussed earlier—to rely predominantly on single-sequence data during training. However, emerging evidence indicates that the integration of multi-sequence MRI, particularly when combined with subtraction imaging techniques, can substantially enhance model performance in BM detection (7).

Although multi-sequence strategies improve diagnostic sensitivity, their clinical applicability is often limited by incomplete imaging protocols in real-world settings (8). To address this issue, the research group led by Darvin Yi introduced a Dropout-based training regimen that randomly masks pulse sequences during model training. This method not only enhances robustness to missing sequences but also preserves high detection accuracy when all sequences are available (9).

For achieving optimal detection performance, deep learning ensemble models have shown considerable promise in effectively integrating multimodal MRI data, demonstrating superior capability in the accurate identification of BM across diverse clinical scenarios (10).

### 2.3 Research on emerging MRI image sequences

The integration of artificial intelligence with novel MRI sequences for model training and validation—building upon established technological foundations—has attracted significant attention within the neuro-oncology and radiology research communities. A notable example is black blood (BB) imaging, an advanced sequence that enhances lesion conspicuity by suppressing intravascular signal, thereby improving contrast between metastases and adjacent vascular structures (11). When coupled with widely adopted architectures such as the 3D U-Net, BB imaging has been shown to deliver superior detection performance and robust generalizability across diverse clinical datasets (12).

### 2.4 Research on small brain metastases

The detection of small BM presents notable imaging challenges attributable to their limited spatial extent and subtle presentation. Key among these are partial volume effects in high-resolution MRI, which can induce signal blurring and reduce overall image clarity. Additionally, smaller BM lesions often exhibit less pronounced contrast enhancement compared to their larger counterparts, complicating differentiation from adjacent vascular structures. Further reducing detectability, lesions situated near blood vessels or dural sinuses are frequently obscured by flow-related artifacts.

Current advances in computer-aided detection (CAD) systems, augmented with DL, demonstrate the potential to overcome these limitations. DL-enhanced CAD can delineate lesion boundaries with high precision, outperforming conventional clinical detection methods in accuracy and reproducibility (13).

### 2.5 Application in CT imaging

Owing to the prolonged acquisition times and limited accessibility of MRI, CT remains widely utilized for the screening of BM. Conventional CT evaluation depends on radiologists performing side-by-side visual assessment of contrast-enhanced (CE) and non-contrast (NECT) scans—a process that is not only labor-intensive but also prone to diagnostic oversights.

Recent advances demonstrate that leveraging both CE and NECT images as model inputs, while minimizing inter-sequence discrepancies, can significantly enhance detection performance while fulfilling the real-time requirements of clinical workflows (14). In a study by Dylan G. Hsu et al., a model trained on paired T1ce and CECT images achieved an overall detection sensitivity of 90%, with sensitivities of 63%−68% for metastases smaller than 3 mm. This represents a marked improvement over models trained on T1ce images alone (15).

## 3 AI differential diagnosis of BM

AI enables high-sensitivity analysis of MRI, CT, and other imaging modalities. It can accurately detect millimeter-scale lesions and extract subtle imaging features—such as ill-defined lesion margins, heterogeneity in enhancement patterns, and perilesional edema distribution—that are challenging to discern with the human eye. These capabilities significantly enhance the ability of radiologists to differentiate between primary gliomas, brain metastases, and other intracranial pathologies. A summary of the relevant research is provided in Table 2.

**Table 2:** Summary of the differentiating model for brain metastases.

**Models**	**ResNet101**	**MFFC-Net**	**ResNet-50**	**CNN**	**IsoSVM**	**MPRM**
Authors	Tariciotti et al.	Liu et al.	Shini et al.	Zahra Riahi Samanii et al.	Luke Pengi et al.	Anthony Lausch et al.
Number of patients	121	1,225	598 + 143	106 + 30	66	19
Exclusion criteria	Image is missing or incomplete; Previous history of intracranial interventions; Multiple lesions	Poor image quality; atypical case	Multiple lesions; Image missing or incomplete; Previous history of intracranial interventions	No specific exclusion criteria were explicitly mentioned	Exclude cases with severe motion artifacts or missing T1 enhancement and T2 FLAIR sequences on MRI	No specific exclusion criteria were explicitly mentioned
Conclusion	Excellent performance in distinguishing PCNSL (AUC: 0.98) from GBM (AUC: 0.90), moderate ability in distinguishing BM (AUC: 0.81)	The model accuracy is 0.920, which is better than that of the radiomics model (0.829) and comparable to that of expert radiologists (0.924).	Performed well in the internal test set (AUC: 0.889) and the external test set (AUC: 0.835), comparable to experienced radiologists.	The CNN model with free water volume fraction (FW-VF) performs excellently (with an accuracy of 93%), outperforming traditional DTI metrics (such as FA and MD) and texture features	In the cross-validation of the retention method, the AUC was 0.81, and the specificity (86.67%) was significantly higher than that of the neuroradiologist's interpretation (19%).	MPRM can predict OS in peritumoral area analysis (sensitivity of 80%, specificity of 100%, and accuracy of 89%, *p* = 0.001)
Limitations	The sample size is relatively small; data from a single center; only the T1Gd sequence was used.	Only use T2 Flair and CE-T1WI; no independent testing group validation has been conducted; the number of doctors participating is limited.	Utilize only 2D analysis; Exclude other types of brain tumors (such as lymphoma); Employ only T2WI and CE-T1WI.	The dataset comes from a single institution; Uneven number of cases; Subdivision analysis not covering different molecular subtypes or primary cancer types	The sample size is relatively small; The processing of mixed pathological specimens may affect the accuracy of the model; The image acquisition parameters and treatment machine have not undergone intensity normalization processing	Small sample size (n=19); Dependent on imaging endpoints; ROI definition may introduce noise; Multi center verification is required

This table provides an overview of current models for discriminating brain metastases from other intracranial lesions. While the development of such diagnostic models has progressed rapidly, future efforts should prioritize multimodal data fusion and the integration of histopathological and genomic information to enhance diagnostic accuracy and clinical applicability.

### 3.1 Distinguishing between BM and GBM

As a common malignant brain tumor, BM exhibits numerous imaging features overlapping with glioblastoma (GBM), posing considerable diagnostic challenges for physicians (16). While conventional radiological characteristics can aid in distinguishing BM from GBM, the use of contrast-enhanced imaging further improves lesion detectability (17). Studies have demonstrated that DL models applied to conventional MRI can support preoperative differentiation between GBM and solitary BM, achieving superior diagnostic accuracy compared to neuroradiologists (18). Moreover, integrating multiparametric MRI to capture spatial tumor heterogeneity has been shown to significantly enhance model performance, with reported sensitivity, specificity, and accuracy reaching 80%, 100%, and 89%, respectively—outperforming traditional single-parameter parametric response mapping (PRM) and tumor volume–based metrics (19). Notably, this approach holds promise for extension to other cancer types and biomarker discovery studies, offering a novel technological pathway for personalized treatment evaluation. Traditionally, tumor classification relies on histopathological biopsy and multiparametric MRI assessment. By exploiting differences in extracellular water content between vasogenic edema and infiltrating tumor tissue—combined with innovative DL techniques designed to characterize the peritumoral microenvironment of GBM and BM—distinctive tumor signatures can be identified to improve diagnostic precision (20).

### 3.2 Distinguish between BM and radiation necrosis

Distinguishing BM from radiation necrosis (RN)—a delayed complication of radiotherapy—remains a significant diagnostic challenge. RN results from necrosis, inflammatory responses, and vascular injury in irradiated brain tissue, typically manifesting months to years after treatment (21). For existing analytical models, the implementation of novel algorithms offers substantial improvements in performance, moving beyond the sole development of new network architectures to enhance differentiation accuracy and clinical utility (22).

### 3.3 Simultaneously distinguish BM from various other brain tumors

The imaging features of GBM subtypes often overlap with those of primary central nervous system lymphoma (PCNSL) and BM, posing considerable challenges in preoperative differential diagnosis. A ResNet101-based model developed by Leonardo Tariciotti et al. demonstrated that incorporating gadolinium-enhanced MRI significantly improves diagnostic accuracy in distinguishing these entities (23). Although these three tumor types share certain conventional MRI characteristics across multiple sequences, they also exhibit distinct radiomic profiles. The extraction and integration of multi-sequence radiomic features through AI can substantially enhance lesion classification and diagnostic precision (24).

## 4 AI-assisted treatment for BM

AI enables the prediction of progression-free survival (PFS) and overall survival (OS) in patients with brain metastases by integrating quantitative imaging features with clinical data. This integrated approach provides a robust foundation for developing personalized treatment strategies and improving prognostic assessment. A summary of the supporting research is presented in Table 3.

**Table 3:** Summary of predictions for brain metastases.

**Models**	**Deep Learning Systems (DLS)**	**Conditional Generative Adversarial Network (cGAN)**	**XGBoost**	**2D Conv-GRU, 3D Conv-GRU, Dmax, radiomics**	**Random Forest (RF)**	**GoldCC, CctoMC, GoldMC**
Authors	Hana Jeongi et al.	Yuhao Yani et al.	Chaofan Lii et al.	Se Jin Choi et al.	Andrei Mouravievi et al.	Luigi Manco et al.
Number of patients	193 + 112	12 + 9	1,933 + 67	194	87	This study is a physical validation and did not utilize patient data.
Exclusion criteria	No brain metastasis; Small cell lung cancer; Received treatment; History of whole brain radiotherapy; There is enhancement of non-brain metastases; Lack of reference standard follow-up data	No specific explanation provided	Two or more primary cancers; Survival time unknown; Age under 20 years old; Male patients with breast cancer; Missing patients	History of whole brain surgery or radiation therapy; Lack of 1 mm thick 3D enhanced T1 weighted image; Visible BM nodules < 5 mm	Cystic metastatic tumor; Non parenchymal metastatic tumor; surgical cavity; received SRS	This study is a physical validation and did not utilize patient data.
Conclusion	The consistency with clinical treatment decisions is 76.8% (81.3% after considering targeted drugs); 95% sensitivity and 81% specificity in high-risk patients	The synthesized CT generated by cGAN showed excellent performance in low field MRI, with a MAE of 70.9 ± 10.4 HU and negligible dosimetric differences; Consistent performance in external validation.	The AUC of XGBoost model predicting 6-month, 1-year, 2-year, and 3-year survival rates are 0.824, 0.813, 0.800, and 0.803, respectively	The 2D Conv GRU model outperforms other models in predicting SRS treatment response, and its accuracy improves with an increasing number of follow-up MRIs.	The integration of radiomics features with clinical features can significantly enhance the accuracy of predicting local failure, particularly the sphericity of the tumor core.	The GoldMC model demonstrated good dose calculation accuracy (≤3% error, gamma pass rate ≥ 95%) across all three types of LINAC.
Limitations	Single center research; The sample size is relatively small; Possible underestimation of the volume of cystic/necrotic lesions; Clinical benefits need to be further validated through long-term results	Small sample size; Limited generalization ability for post-operative abnormal anatomical structures; Further validation is needed to confirm its applicability in a multi center environment	The SEER database lacks data on disease recurrence or subsequent metastasis sites; Racial differences may limit the universality of the model; Lack of detailed information on brain metastasis treatment	No external verification has been conducted; Small sample size; The ground truth values are mainly based on clinical and radiological information; The preprocessing and segmentation processes are complex	The sample size is relatively small; The endpoint of the lesion is mainly based on imaging criteria rather than pathological confirmation; The importance of FLAIR features is relatively low	Model optimization requires a compromise to adapt to three LINACs; CctoMC performs slightly better in some complex scenarios, but the difference is minimal.

This table is a summary of the prediction models for brain metastases. The MC algorithm has excellent physical accuracy, but its computing speed cannot meet the real-time clinical needs. Currently, research is mostly focused on model construction and technical validation, lacking evidence embedded in actual diagnosis and treatment processes and evaluation of its impact on final treatment outcomes.

### 4.1 Optimization of treatment plan

Among brain tumors, brain metastases (BM) are recognized as one of the most aggressive entities. Their pronounced morphological heterogeneity, however, complicates clinical efforts to optimize treatment dosing and intensity. Early subtype differentiation of BM can facilitate more tailored therapeutic strategies, thereby improving disease control and helping preserve functional brain tissue (25). Rapid advances in AI-driven BM segmentation have enabled classification algorithms based on segmentation network architectures to enhance patient stratification and significantly reduce diagnostic and therapeutic timelines (26).

Owing to their distinct imaging principles, CT and MRI capture complementary characteristics of BM, offering a more comprehensive diagnostic basis and supporting more precise radiation planning with reduced marginal failures. Nevertheless, the extended acquisition times associated with combined CT and MRI protocols may delay treatment initiation, potentially compromising clinical outcomes. There is growing interest in reducing dependency on multi-modal imaging—beginning at the acquisition stage—to maintain radiotherapy accuracy without sacrificing detection efficacy (27).

### 4.2 Predicting treatment response and local failure

Stereotactic radiosurgery (SRS) offers significant benefits for patients with BM, including local tumor control, symptomatic relief, and potential improvement in overall survival. Nevertheless, challenges persist in optimizing drug selection, dosage individualization, and treatment parameter configuration, underscoring the need for AI-based prognostic tools. Integrating radiomic features from BM with ML enables assessment of local recurrence risk following SRS. Beyond conventional predictors, tumor core sphericity has emerged as an independent prognostic factor that may further enhance predictive accuracy (28).

The Monte Carlo (MC) technique represents the most accurate method for dose calculation and optimization in radiotherapy and serves as a key tool for validating treatment plan accuracy. However, its clinical adoption has been limited by computationally intensive processes. In a systematic clinical validation across multiple matched linear accelerators (LINACs), Luigi Manco et al. demonstrated that the optimized GoldMC model achieved agreement within 3% between calculated and measured point doses, underscoring the physical precision of MC algorithms in managing electron transport in heterogeneous media (29). Despite this technical advancement, the model has not yet been integrated into real-world clinical workflows or evaluated for its impact on final patient outcomes.

Furthermore, the incorporation of longitudinal imaging data into predictive models can substantially improve prognostic precision. Predictive performance continues to enhance with an increasing number of follow-up scans, enabling more dynamic and personalized risk assessment (30).

### 4.3 Survival prediction

AI-powered dynamic tracking technology enables quantitative assessment of changes in lesion volume following radiotherapy or targeted therapy. By detecting and amplifying subtle differences in metabolic activity within affected regions, this approach effectively differentiates between radiation necrosis and tumor recurrence, providing critical data to guide adjustments in clinical management strategies. When integrated with patient-specific clinical characteristics, algorithm-enhanced Cox regression models contribute to the prediction of survival outcomes. Several established prognostic factors—such as the association between higher socioeconomic status and prolonged survival, as well as the benefits of surgical intervention in certain molecular subtypes—further refine these predictions (31).

Moreover, multimodal diagnostic models that incorporate primary tumor history, serum biomarkers, and genomic profiling can assist in identifying the origin of metastases, thereby supporting clinical decision-making. From an implementation perspective, AI systems are capable of autonomously screening electronic health records within seconds, maintaining high diagnostic accuracy while significantly reducing the cognitive burden on clinicians.

## 5 Discussion

### 5.1 Limitations of AI

Although AI has shown significant promise in the imaging-based management of brain metastases, several key limitations impede its broad integration into clinical practice:

Methodological Constraints: Many studies exhibit inherent data biases—such as retrospective designs, limited sample sizes, and inconsistent annotation standards—which substantially compromise the reliability and generalizability of the reported outcomes.

Computational Complexity in Longitudinal Analysis: While longitudinal tracking has been explored, accurate image registration across time points remains challenging, particularly with interval development of new lesions or substantial changes in existing lesion morphology. Reliable and automated volume monitoring remains computationally intensive and technically demanding, requiring further optimization (5, 10, 30).

Unclear Impact on Clinical Decision-Making: Although AI is designed to augment rather than replace clinical judgment, critical issues such as accountability in misdiagnosis, effective human–AI collaboration, and the ethical implications of automated decision support have not been sufficiently addressed.

Clinical Integration and Workflow Barriers: Most existing evidence stems from retrospective studies, with a notable absence of prospective clinical validation. It remains uncertain whether AI-assisted workflows can tangibly improve patient outcomes in real-world settings, underscoring the need for large-scale prospective trials.

### 5.2 The advantages of AI

Research on AI in BM imaging has advanced considerably, with its core clinical value manifested in three key areas: precision detection, efficient segmentation, and intelligent decision support.

DL-based detection algorithms enable rapid identification of millimeter-scale metastases, significantly improving sensitivity and reducing missed diagnoses. Multimodal image fusion further enhances model performance in complex clinical scenarios—particularly in differentiating radiation necrosis from tumor recurrence—thereby providing robust support for diagnostic decision-making.

Treatment planning and prognostic evaluation have also been optimized through automated segmentation tools that deliver submillimeter accuracy in target delineation for SRS. Supplemented by synthetic CT generation via GANs, AI reduces reliance on conventional CT and streamlines the radiotherapy workflow. Furthermore, AI-enhanced models that integrate radiomic features with clinical variables improve survival prediction accuracy, establish a basis for personalized therapy, and offer clinicians multiple evidence-based options during strategy formulation.

AI also promotes non-invasive “virtual biopsy” by extracting subtle imaging features beyond human visual perception, allowing detailed assessment of metastatic phenotypes. Integrating radiomics with PET-MRI multimodal characteristics further enables discrimination between GBM and solitary BM, potentially reducing the need for invasive craniotomy biopsies and advancing patient-centric diagnostic pathways (32).

Despite this promising trajectory, clinical integration of AI in BM management continues to face challenges such as data heterogeneity and model interpretability. Future efforts should prioritize multicenter validation, real-world performance monitoring, and cross-disciplinary collaboration—including genomic data integration—to ultimately achieve a closed-loop intelligent system spanning from imaging diagnosis to therapeutic intervention.

### 5.3 Future directions

Data Standardization and High-Quality Multicenter Databases: Multicenter studies with large datasets represent the gold standard for validating the clinical applicability of AI models (6, 12). Establishing unified image acquisition protocols and annotation standards is essential to facilitate the development of large-scale, prospective, and multicenter databases. Furthermore, the adoption of privacy-preserving computational techniques—such as federated learning—can enable collaborative multicenter modeling while ensuring stringent protection of patient data privacy.

Characterization of Tumor Microenvironment and Biological Behavior: Analysis of peritumoral microenvironmental features has revealed distinct imaging characteristics between glioblastoma and brain metastases, enhancing our understanding of their divergent growth and dissemination patterns. When integrated with AI-driven imaging genomics, these features can bridge radiographic appearances with gene expression profiles, enabling non-invasive assessment of molecular tumor characteristics (2, 20).

Precision Radiotherapy Planning: AI-based automatic segmentation of tumor target volumes reduces inter-observer variability in delineation and improves radiotherapy precision. The incorporation of Monte Carlo algorithms shows strong potential in enhancing dose calculation accuracy during treatment delivery (29). Additionally, deep learning-generated synthetic CT images from low-field MRI offer a pathway toward purely MRI-guided radiotherapy, which may eliminate the need for CT simulation, streamline workflows, and minimize systematic errors (27).

Methodological Robustness: The nnU-Net framework has become a benchmark in medical image segmentation by automatically adapting to diverse datasets and optimizing architecture configurations, effectively balancing generalizability and task-specific performance (6). Meanwhile, ensemble learning techniques help mitigate overfitting risks and enhance model robustness and accuracy, proving particularly valuable when analyzing complex medical imaging data (10).

## References

[1] Lamba N Wen PY Aizer AA. Epidemiology of brain metastases and leptomeningeal disease. Neuro Oncol. (2021) 23:1447–56. 10.1093/neuonc/noab101 33908612 PMC8408881

[2] Gillespie CS Mustafa MA Richardson GE Alam AM Lee KS Hughes DM. Genomic alterations and the incidence of brain metastases in advanced and metastatic nsclc: a systematic review and meta-analysis. J Thorac Oncol. (2023) 18:1703–13. 10.1016/j.jtho.2023.06.017 37392903

[3] Zhou Z Sanders JW Johnson JM Gule-Monroe M Chen M Briere TM. MetNet: computer-aided segmentation of brain metastases in post-contrast T1-weighted magnetic resonance imaging. Radiother Oncol. (2020) 153:189–96. 10.1016/j.radonc.2020.09.016 32937104

[4] Zhou Z Qiu Q Liu H Ge X Li T Xing L. Automatic detection of brain metastases in T1-weighted construct-enhanced MRI using deep learning model. Cancers. (2023) 15:4443. 10.3390/cancers15184443 37760413 PMC10526374

[5] Hammer Y Najjar W Kahanov L Joskowicz L Shoshan Y. Two is better than one: longitudinal detection and volumetric evaluation of brain metastases after Stereotactic Radiosurgery with a deep learning pipeline. J Neurooncol. (2024) 166:547–55. 10.1007/s11060-024-04580-y 38300389 PMC10876809

[6] Yoo Y Gibson E Zhao G Re TJ Parmar H Das J. Extended nnU-net for brain metastasis detection and segmentation in contrast-enhanced magnetic resonance imaging with a large multi-institutional data set. Int J Radiat Oncol. (2025) 121:241–9. 10.1016/j.ijrobp.2024.07.2318 39059508 PMC13426310

[7] Li R Guo Y Zhao Z Chen M Liu X Gong G. MRI-based two-stage deep learning model for automatic detection and segmentation of brain metastases. Eur Radiol. (2023) 33:3521–31. 10.1007/s00330-023-09420-7 36695903

[8] Jünger ST Hoyer UCI Schaufler D Laukamp KR Goertz L Thiele F. Fully automated MR detection and segmentation of brain metastases in non-small cell lung cancer using deep learning. Magn Reson Imaging. (2021) 54:1608–22. 10.1002/jmri.27741 34032344

[9] Yi D Grøvik E Tong E Iv M Emblem KE Nilsen LB. MRI pulse sequence integration for deep-learning-based brain metastases segmentation. Med Phys. (2021) 48:6020–35. 10.1002/mp.15136 34405896

[10] Machura B Kucharski D Bozek O Eksner B Kokoszka B Pekala T. Deep learning ensembles for detecting brain metastases in longitudinal multi-modal MRI studies. Comput Med Imaging Graphics. (2024) 116:102401. 10.1016/j.compmedimag.2024.102401 38795690

[11] Chaganti J Woodford H Tomlinson S Dunkerton S Brew B. Black blood imaging of intracranial vessel walls. Pract Neurol. (2021) 21:101–7. 10.1136/practneurol-2020-002806 33376151

[12] Park YW Park JE Ahn SS Han K Kim N Oh JY. Deep learning-based metastasis detection in patients with lung cancer to enhance reproducibility and reduce workload in brain metastasis screening with MRI: a multi-center study. Cancer Imaging. (2024) 24:32. 10.1186/s40644-024-00669-9 38429843 PMC10905821

[13] Fairchild AT Salama JK Wiggins WF Ackerson BG Fecci PE Kirkpatrick JP. A deep learning-based computer aided detection (CAD) system for difficult-to-detect brain metastases. Int J Radiat Oncol. (2023) 115:779–93. 10.1016/j.ijrobp.2022.09.068 36289038

[14] Takao H Amemiya S Kato S Yamashita H Sakamoto N Abe O. Deep-learning single-shot detector for automatic detection of brain metastases with the combined use of contrast-enhanced and non-enhanced computed tomography images. Eur J Radiol. (2021) 144:110015. 10.1016/j.ejrad.2021.110015 34742108

[15] Hsu DG Ballangrud Å Shamseddine A Deasy JO Veeraraghavan H Cervino L. Automatic segmentation of brain metastases using T1 magnetic resonance and computed tomography images. Phys Med Biol. (2021) 66:175014. 10.1088/1361-6560/ac1835 34315148 PMC9345139

[16] Liu Y Li T Fan Z Li Y Sun Z Li S. Image-based differentiation of intracranial metastasis from glioblastoma using automated machine learning. Front Neurosci. (2022) 16:855990. 10.3389/fnins.2022.855990 35645718 PMC9133479

[17] de Causans A Carré A Roux A Tauziède-Espariat A Ammari S Dezamis E. Development of a machine learning classifier based on radiomic features extracted from post-contrast 3D T1-weighted MR images to distinguish glioblastoma from solitary brain metastasis. Front Oncol. (2021) 11:638262. 10.3389/fonc.2021.638262 34327133 PMC8315001

[18] Shin I Kim H Ahn SS Sohn B Bae S Park JE. Development and validation of a deep learning–based model to distinguish glioblastoma from solitary brain metastasis using conventional MR images. AJNR Am J Neuroradiol. (2021) 42:838–44. 10.3174/ajnr.A7003 33737268 PMC8115383

[19] Lausch A Yeung TP Chen J Law E Wang Y Urbini B. A generalized parametric response mapping method for analysis of multi-parametric imaging: a feasibility study with application to glioblastoma. Med Phys. (2017) 44:6074–84. 10.1002/mp.12562 28875538

[20] Samani ZR Parker D Wolf R Hodges W Brem S Verma R. Distinct tumor signatures using deep learning-based characterization of the peritumoral microenvironment in glioblastomas and brain metastases. Sci Rep. (2021) 11:14469. 10.1038/s41598-021-93804-6 34262079 PMC8280204

[21] Mayo ZS Billena C Suh JH Lo SS Chao ST. The dilemma of radiation necrosis from diagnosis to treatment in the management of brain metastases. Neuro Oncol. (2024) 26:S56–65. 10.1093/neuonc/noad188 38437665 PMC10911797

[22] Peng L Parekh V Huang P Lin DD Sheikh K Baker B. Distinguishing true progression from radionecrosis after stereotactic radiation therapy for brain metastases with machine learning and radiomics. Int J Radiat Oncol. (2018) 102:1236–43. 10.1016/j.ijrobp.2018.05.041 30353872 PMC6746307

[23] Tariciotti L Caccavella VM Fiore G Schisano L Carrabba G Borsa S. A deep learning model for preoperative differentiation of glioblastoma, brain metastasis and primary central nervous system lymphoma: a pilot study. Front Oncol. (2022) 12:816638. 10.3389/fonc.2022.816638 35280801 PMC8907851

[24] Liu X Liu J. Aided diagnosis model based on deep learning for glioblastoma, solitary brain metastases, and primary central nervous system lymphoma with multi-modal MRI. Biology. (2024) 13:99. 10.3390/biology13020099 38392317 PMC10887006

[25] Moreau NN Valable S Jaudet C Dessoude L Thomas L Hérault R. Early characterization and prediction of glioblastoma and brain metastasis treatment efficacy using medical imaging-based radiomics and artificial intelligence algorithms. Front Oncol. (2025) 15:1497195. 10.3389/fonc.2025.1497195 39949753 PMC11821606

[26] Jeong H Park JE Kim N Yoon S-K Kim HS. Deep learning-based detection and quantification of brain metastases on black-blood imaging can provide treatment suggestions: a clinical cohort study. Eur Radiol. (2023) 34:2062–71. 10.1007/s00330-023-10120-5 37658885 PMC10873231

[27] Yan Y Kim JP Nejad-Davarani SP Dong M Hurst NJ Jr Zhao J. Deep learning-based synthetic computed tomography for low-field brain magnetic resonance-guided radiation therapy. Int J Radiat Oncol. (2025) 121:832–43. 10.1016/j.ijrobp.2024.09.046 39357787 PMC11875202

[28] Mouraviev A Detsky J Sahgal A Ruschin M Lee YK Karam I. Use of radiomics for the prediction of local control of brain metastases after stereotactic radiosurgery. Neuro Oncol. (2020) 22:797–805. 10.1093/neuonc/noaa007 31956919 PMC7283017

[29] Manco L Vega K Maffei N Gutierrez MV Cenacchi E Bernabei A. Validation of RayStation Monte Carlo dose calculation algorithm for multiple LINACs. Physica Medica. (2023) 109:102588. 10.1016/j.ejmp.2023.102588 37080156

[30] Cho SJ Cho W Choi D Sim G Jeong SY Baik SH. Prediction of treatment response after stereotactic radiosurgery of brain metastasis using deep learning and radiomics on longitudinal MRI data. Sci Rep. (2024) 14:11085. 10.1038/s41598-024-60781-5 38750084 PMC11096355

[31] Li C Liu M Zhang Y Wang Y Li J Sun S. Novel models by machine learning to predict prognosis of breast cancer brain metastases. J Transl Med. (2023) 21:404. 10.1186/s12967-023-04277-2 37344847 PMC10286496

[32] Cao X Tan D Liu Z Liao M Kan Y Yao R. Differentiating solitary brain metastases from glioblastoma by radiomics features derived from MRI and 18F-FDG-PET and the combined application of multiple models. Sci Rep. (2022) 12:5722. 10.1038/s41598-022-09803-8 35388124 PMC8986767

